# A Flight Test of the Strapdown Airborne Gravimeter SGA-WZ in Greenland

**DOI:** 10.3390/s150613258

**Published:** 2015-06-05

**Authors:** Lei Zhao, René Forsberg, Meiping Wu, Arne Vestergaard Olesen, Kaidong Zhang, Juliang Cao

**Affiliations:** 1 College of Mechatronics Engineering and Automation, National University of Defense Technology, Changsha 410073, China; E-Mails: zl_nudt@yahoo.com (L.Z.); kdzhang@263.net (K.Z.); cjl.nudt@gmail.com (J.C.); 2 National Space Institute, Technical University of Denmark, Copenhagen Ø 2800, Denmark; E-Mails: rf@space.dtu.dk (R.F.); avo@space.dtu.dk (A.V.O.)

**Keywords:** gravity data, strapdown airborne gravimeter, inertial navigation system, finite impulse response filter, global positioning system

## Abstract

An airborne gravimeter is one of the most important tools for gravity data collection over large areas with mGal accuracy and a spatial resolution of several kilometers. In August 2012, a flight test was carried out to determine the feasibility and to assess the accuracy of the new Chinese SGA-WZ strapdown airborne gravimeter in Greenland, in an area with good gravity coverage from earlier marine and airborne surveys. An overview of this new system SGA-WZ is given, including system design, sensor performance and data processing. The processing of the SGA-WZ includes a 160 s length finite impulse response filter, corresponding to a spatial resolution of 6 km. For the primary repeated line, a mean r.m.s. deviation of the differences was less than 1.5 mGal, with the error estimate confirmed from ground truth data. This implies that the SGA-WZ could meet standard geophysical survey requirements at the 1 mGal level.

## 1. Introduction

Airborne gravimetry, which can fill the gap between satellite observations and terrestrial gravity field measurements at a spatial resolution of several kilometers over large areas, is an efficient way to map the Earth’s gravity field [1,2,3,4,5]. There are two main categories of airborne gravimeters for scalar gravimetry, either stabilized platform systems, or strapdown systems [6]. Most systems used operationally up to now are of the first type, based on either modified marine gravimeters or modified inertial systems with a physical gyro-stabilized platform. The first stabilized platform airborne gravimetric system was made by LaCoste, and already used for airborne tests in the late 1960s [7,8]. However, airborne gravimeters didn’t see significant development until there was the necessary improvement of position, velocity and acceleration determination by the global positioning system (GPS) in the late 1980s [4,9]. 

So far, several airborne gravimeters have been used to survey the gravity field, such as the Lacoste and Romberg air-sea gravimeter (LCR), Russian Chekan-AM, GT-1A, Sanders AirGrav and various strapdown inertial scalar gravimetry (SISG) tests [6,10,11,12,13,14,15]. Airborne gravimeters on a stabilized platform typically have higher accuracy and less drift, with strapdown airborne gravimeters having the advantage of being a simpler structure with light weight, small size, low cost, low power consumption and easy operation [6,16]. Besides, strapdown airborne gravimeters can be used to implement vector gravimetry [17,18,19]. Therefore, airborne gravimeters based on strapdown the inertial navigation system (INS) has been seen many development efforts for airborne gravimetry applications for many years.

The first Chinese airborne scalar gravimeter based on strapdown INS (called SGA-WZ) was made by the National University of Defense Technology (NUDT) in 2008. Several tests have been carried out in China to determine the feasibility and to assess the accuracy of this system [20,21,22]. This paper shows results of a joint Technical University of Denmark (DTU) Space-NUDT test of SGA-WZ in Greenland in 2012 under rough field conditions, repeating earlier LCR flights. A comparison between the results of these two systems will be shown in the end.

## 2. Principle of Strapdown Airborne Gravimeter

According to Newton’s Second Law, gravity can be extracted from a combination of accelerometers and a kinematic navigation system, such as GPS. In the inertial reference frame (i-frame), the definitive equation for gravimetry could be written as:

(1) $$g^{i}={\ddot{r}}^{i}-f^{i}$$

where $g^{i}$ is gravitational acceleration, ${\ddot{r}}^{i}$ is the acceleration of the aircraft and $f^{i}$ are the specific force sensed by accelerometers in i-frame. Transforming the equation into the navigation frame (n-frame), it becomes:

(2) $$g^{n}={\dot{v}}_{e}^{n}-C_{b}^{n}f^{b}+(2\omega_{ie}^{n}+\omega_{en}^{n})\times v_{e}^{n}$$

where ${\dot{v}}_{e}^{n}$ and $v_{e}^{n}$ are the acceleration and velocity of vehicle with respect to the Earth, $f^{b}$ is the specific force measured by triad of accelerometers of a strapdown INS in body frame (b-frame), $C_{b}^{n}$ is the direct cosine matrix from b-frame to the n-frame. $g^{n}$ is the gravity vector, $\omega_{ie}^{n}$ is the rotation rate of the Earth with respect to the n-frame. $\omega_{en}^{n}$ is angular rate of the n-frame with respect to the Earth frame(e-frame), expressed in the n-frame. 

The gravity vector can be written as the sum of the normal gravity vector γ and the gravity disturbance vector δg. Thus, the measurement model for airborne gravimetry is given as:

(3) $$\delta g^{n}={\dot{v}}_{e}^{n}-C_{b}^{n}f^{b}+(2\omega_{ie}^{n}+\omega_{en}^{n})\times v_{e}^{n}-\gamma^{n}$$

For scalar gravimeter only the vertical quantity of the gravity (the third term of Equation (3)) is of interest. When written explicitly it is:

(4) $$\delta g_{D}={\dot{v}}_{D}-f_{D}+(2\omega_{ie}\cdot \cos\varphi +\frac{v_{E}}{R_{N}+h})\cdot v_{E}+\frac{v_{N}^{2}}{R_{M}+h}-\gamma$$

where subscripts N,E,D stand for North, East, Down in a local-level ellipsoidal frame(n-frame). $v_{N}$,$v_{E}$ and $v_{D}$ represent the north, east and down elements of the aircraft velocity respectively. $f_{D}$ is the down component of specific force in n-frame. γ is the down component of the normal gravity $\gamma^{n}$. φ is the latitude in n-frame, h is the height of the flight. $R_{M}$ and $R_{N}$ are the prime vertical and meridian radii of curvature respectively. Denote $\delta a_{E}$ is the sum of the third and fourth terms:

(5) $$\delta a_{E}=(2\omega_{ie}\cdot \cos\varphi +\frac{v_{E}}{R_{N}+h})\cdot v_{E}+\frac{v_{N}^{2}}{R_{M}+h}$$

$\delta a_{E}$ is also called the Eötvös correction which is the correction for velocity relative to the Earth during a measurement activity [3,23].

Equation (4) shows the basic principle of a strapdown inertial scalar gravimeter (SISG) and the gravity disturbance $\delta g_{D}$ are expressed in terms of observed quantities. When using a merged INS/GPS as an airborne gravity system, the vehicle kinematic quantities including the latitude (φ), the flight height (h), the north and east component of velocity of the aircraft ($v_{N}$ and $v_{E}$), the down component of acceleration of the aircraft (${\dot{v}}_{D}$) and the normal gravity γ can be obtained from GPS position and velocity results. The down component of the specific force ($f_{D}$) is transformed from $f^{b}$ by transformation matrix $C_{b}^{n}$. Thus, the gravity disturbance $\delta g_{D}$ will be estimated from the output of INS and GPS. More details on the basic principle of SISG can be found in Wei and Schwarz [3]. Using SGA-WZ as an example, the implementation of Equation (4) will be given in the next section.

## 3. System Description

SGA-WZ, ranked as the first Chinese strapdown airborne gravimeter, was made by NUDT in 2008. This new airborne gravimetric system is based on a strapdown INS and phase Differential GPS (DGPS). In this section, the description of SGA-WZ is given including the system structure, sensor performance and data processing.

### 3.1. Structure of SGA-WZ

**Figure 1:**
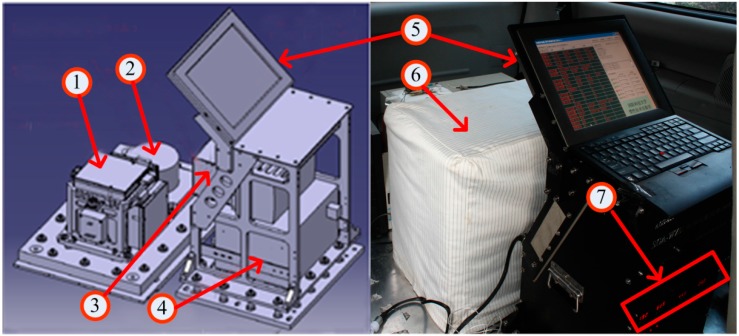
Structure of SGA-WZ. In the left figure, it is the 3D model of SGA-WZ without any shells; the right figure shows the working state of the system.

Figure 1 shows the whole structure of SGA-WZ which can be divided into two main parts: Sensors Box and Control Box. 

The Sensor Box consists of a strapdown INS in which there are three navigation-grade Ring Laser Gyroscopes (RLG) and three quartz flexibility accelerometers (No. 1 in the figure), one triad of higher accuracy accelerometers (No. 2), and one ‘coat’ as an insulation shell to keep temperature steady in the box (No. 6). It also can be seen in Figure 1 that the triad of accelerometers is not installed inside but side by side with the set of RLGs. Either triad of accelerometers can be used in inertial navigation computation of SGA-WZ, however, because dither RLGs could make high frequent vibration which will decrease the accuracy and resolution of the accelerometers, in this study only the higher accuracy triad of accelerometers was applied for data processing.

The Control Box is composed of a device for data logger (No. 3), an uninterruptible power supply (UPS) which can supply uninterrupted power for the whole system (No. 4), a computer for recording all data and monitoring instruments running status (No. 5) and several thermostats used to regulate temperature inside of the Sensor Box (No. 7). All inertial data are recorded at a rate of 2 kHz while GPS data are recorded from a dual frequency receiver at a rate of 1 or 2 Hz. The rate of recording GPS data can be changed according to different demands.

NUDT designed and manufactured the framework of SGA-WZ, the measuring circuits and UPS control system, assembled the strapdown INS and the triad of higher accuracy accelerometers, and developed the data recording software and processing program. All of these works make it easier to localize the source of a problem and fix it in a campaign.

### 3.2. Performance of Sensors

The performance of sensors is the key criterion of an airborne gravimeter, and these sensors include RLGs and accelerometers which measure angular motion and translation motion respectively. SGA-WZ has three RLGs with a stability of ±0.004°/h and random noise of 0.002°/$\sqrt{\text{h}}$, which satisfies the demand of an airborne scalar gravimeter with a level of 1 mGal/1 km in ideal conditions and the average flight speed is under 60 km/h [16]. 

Compared to RLGs, the performance of accelerometers is more critical for airborne gravimeter [21]. Without thermal control, accelerometers in SGA-WZ have a stability of ±0.2 mGal in four hours, random noise of 5$\text{mGal/}\sqrt{\text{Hz}}$ and scale factor uncertainty of ±30 ppm. Since the temperature coefficient of these accelerometers is relatively high, a precise thermal control at the level of 0.02 °C is designed in SGA-WZ to improve the stability of accelerometers to a level of 0.5 mGal/day [21]. Moreover, a static test was carried out to check the long-term reliability of the system in a laboratory. The result shows that SGA-WZ can work well for a long time [21].

The performance of the sensors shows that SGA-WZ can perform the work of obtaining gravity data at the level of 1 mGal/1 km in ideal conditions. However, in a real campaign, all kinds of dynamic motions as well as the aircraft engines cause significant noise, which consequently contaminates the accuracy of the gravity sensors and decreases the resolution and accuracy of final measurement results.

### 3.3. Data Processing

**Figure 2:**
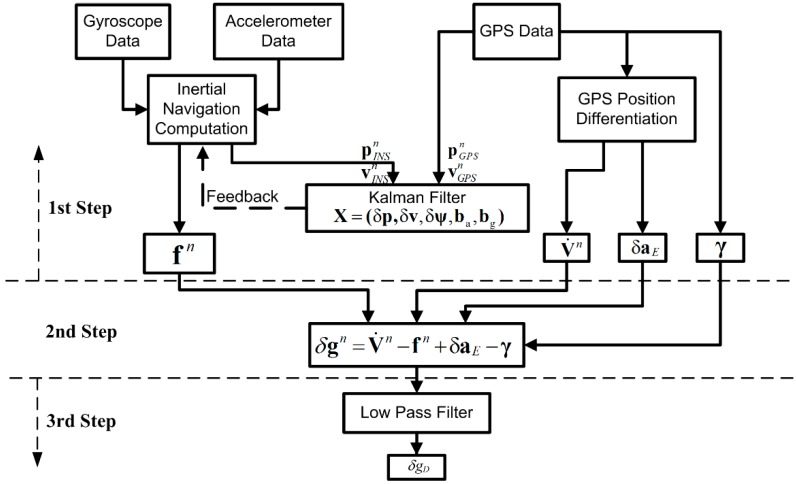
Configuration of data processing for SGA-WZ. The estimated Kalman filter states are position, velocity, attitude, and gyro and accelerometer biases.

To extract the airborne gravity disturbance from the difference between the specific force vector and the GPS-derived acceleration vector, the processing can be divided into three distinct steps. Figure 2 shows the configuration of these steps for data processing. Prior to implementing this process the output of sensors are resampled to 100 Hz, as a higher sampling rate doesn’t matter for airborne gravimetry. Position and velocity of the aircraft are processed with the Waypoint GrafNav software, and the accelerations are obtained by double differentiation of the GPS positions. To keep the focus on gravity estimation, more details of the GPS data processing are not included in this study.

As shown in Figure 2, in the first step, on one hand, a traditional INS/GPS integration approach, where local gravity variability is not taken into account, is used to compute the attitude, position, and velocity of the airborne gravity system. In this step, accelerometer biases and drift rates of the INS are estimated, for correcting the measurements before the gravity estimation. To estimate the errors, including biases and drifts of the inertial sensors, the Strapdown INS/GPS integrated navigation computation was implemented with a conventional 15-state Kalman Filter (KF). In the INS/GPS KF, the INS systematic and stochastic errors are determined by constructing error models. Since the sensors of SGA-WZ have good performance, based on sensors’ calibration these error models can be written in following equation:

(6) $$\delta e_{m}=b_{m}+w_{m}$$

where δe represents the sensor error, b represents the sensor bias , w represents the white noise of the sensor. m specifies on accelerometer or gyroscope. Then, the system and observation model for the INS is represented as follows:

(7) $$\begin{array}{l} x_{k+1}=\Phi_{k+1,k}x_{k}+G_{k}w_{k}\\ y_{k+1}=H_{k+1}x_{k+1}+v_{k+1} \end{array}$$

where $x_{k+1}$ is the system error state vector to be estimated at time $t_{k+1}$, $\Phi_{k+1,k}$ is the system state transition matrix, $w_{k}$ is the vector of the system input random noise, $G_{k}$ is the coefficient matrix associated with the system input noise, $y_{k+1}$ is the vector of the system observations at time $t_{k+1}$, $H_{k+1}$ is the design matrix relating the system observations to the system error states, and $v_{k+1}$ is the vector of update measurements random noise. For a 15-state KF, the system error state vector is $\left\{\delta p,\delta v,\delta \text{ψ},\delta b_{a}^{b},\delta b_{g}^{b}\right\}$ which represent the error of 3D position, 3D velocity, 3D attitude, 3D accelerometer biases and 3D gyro biases respectively. The implementation of KF can be found in [24]. At last, compensation for these errors is performed using the KF output. The result of this inertial navigation computation loop $f^{n}$ is the second term in Equation (2). One the other hand, the Eötvös correction (third term in Equation (4)) and normal gravity γ (last term in Equation (4)) is obtained from GPS position and velocity results. 

In the second step, according to the Equation (4), the gravity disturbance at the flight height is estimated from the direct difference between the measured specific force and the vehicle acceleration in the n-frame, after applying the sensor bias corrections estimated in the first step. Although the vertical accelerometer bias and gravity disturbances are strongly correlated, this two-step procedure seems to function well, with the improvement coming both from the different noise correlation assumptions, and decoupling through the natural phugoid motion of the aircraft. A lever-arm correction is used to transfer the INS and GPS measurements into a common system.

In the last step, low-pass filter is applied to reduce the measurement noise in the estimated gravity disturbance. Airborne gravity measurements are made in a very dynamic environment, which results in extremely large noise in the data at the high frequencies. In general, there are two classical low-pass filters: finite impulse response (FIR) and infinite impulse response (IIR) filters. For SGA-WZ, a FIR is designed to extract the gravity signal from the measurement data. The cut-off filter length is determined by the target resolution. In this study, for example, since the minimum half wavelength of the reference LCR airborne gravity data is about 6 km and the average speed of the flight is around 70 m/s, a 160 s length filter is used for low pass filtering. This means the cut-off frequency is 1/160 Hz.

## 4. Test Description

In August 2012, an airborne gravimetry test of SGA-WZ was carried out in central East Greenland, in cooperation between DTU Space and NUDT. In the test flights were both made over very rough mountain and fjord region, with gravity anomalies in excess of several 100 s of mGal, and in the more benign offshore environment. All flights were done at constant elevation flights, with quite large changes in ambient wind and turbulence conditions. The goal of this test is to evaluate repeatability as well as accuracy of the new airborne gravimeter in arctic region.

**Figure 3:**
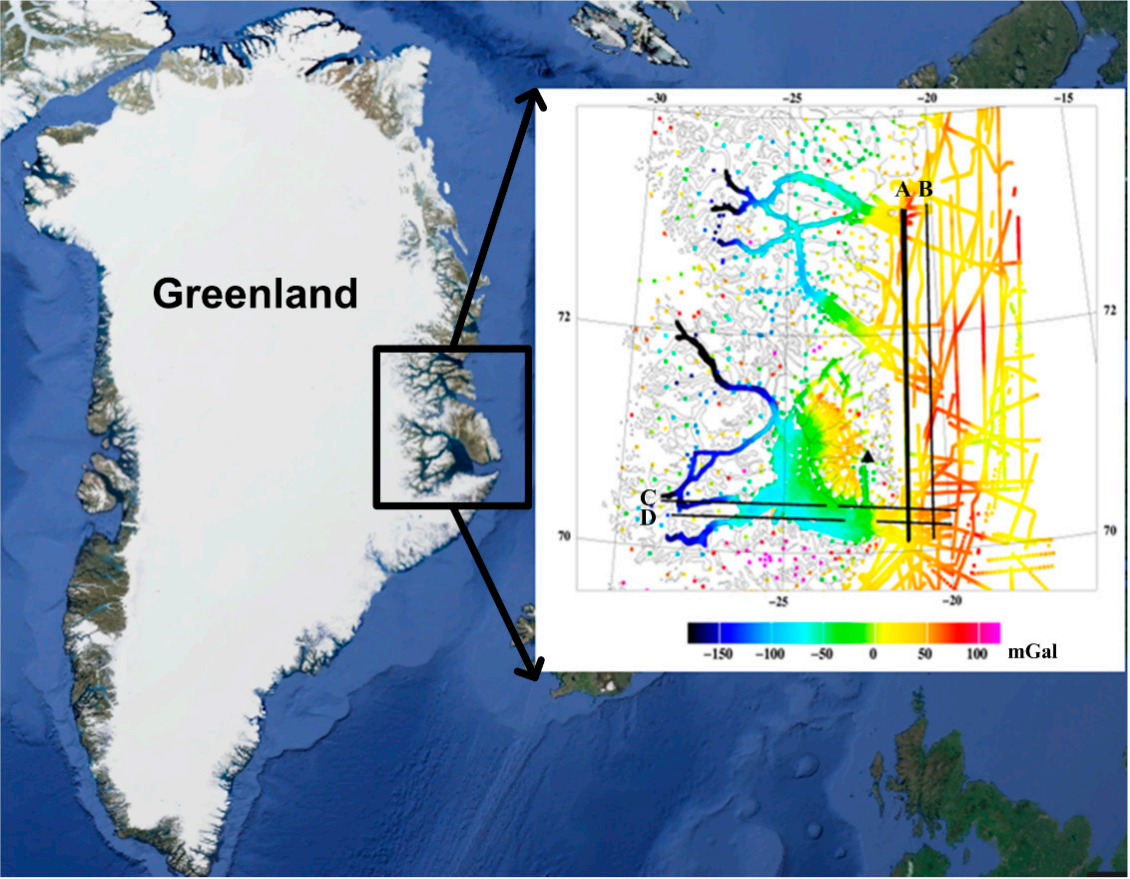
Flight profiles in Greenland test and existing ground, marine and airborne coverage of the region. The four flight profiles shown in black (the primary line A is in bold) were designed in central East Greenland. Breaks in line C and D are due to altitude changes. The colors show the free-air anomalies.

The SGA-WZ was installed in a Nordlandair Twin Otter in Iceland, and data were as collected well during transit flights, and over as a separate lidar flight over the Snæfellsjökull glacier in Iceland. Only the marine data offshore Greenland are reported in this paper. In order to strengthen the reliability of the Greenland test, two GPS receivers were located near the main airport of Constable Point, serving as master station (shown in Figure 3 with a black triangle) for computing the high accuracy position and velocity of the aircraft, and two extra GPS receivers were installed in the plane. All of the GPS receivers were set to record data at a rate of 1 Hz. With the used GrafNav software position errors were typical at the 5–10 cm level, as confirmed from repeated DTU Space lidar campaigns.

There were four flight profiles designed for this test (seen in Figure 3). The two marine lines from north to south (line A in a bold black line and line B separately from left to right) were repeated, and were in a region with relatively good ground and marine data coverage; the other two one-pass W-E profiles (line C and D) are crossing over the southern mountain areas and the 1000 m+ deep fjord of the Scoresbysund system. The average flight speed was about 250 km/h (135 knots).The flight altitude was approximately 360 m above sea-level for line A and B, and over 2000 m for line C and D. Besides, there were breaks in line C and D due to altitude changes.

In order to check the external accuracy of this test, former marine and airborne gravity data around this area was used. The most of the primary marine line had, however, been flown in 2001 as well with a Lacoste and Romberg S-type gravimeter [25]. These data with the resolution of around 6 km, augmented with marine data, were therefore used as “ground truth” for the SGA-WZ test.

## 5. Results and Analysis

**Figure 4:**
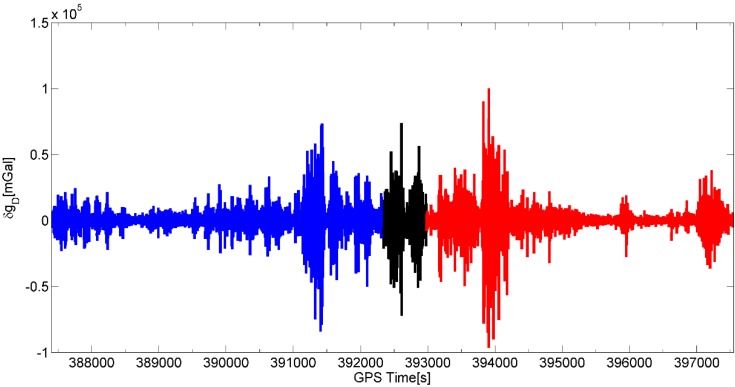
Raw gravity measurements (INS-GPS differences) along primary repeated flight A. The northbound flight is shown in blue while the southbound one is marked in red. The black curve between time 392,000 s and 393,000 s represents a 360° turn of the aircraft.

**Figure 5:**
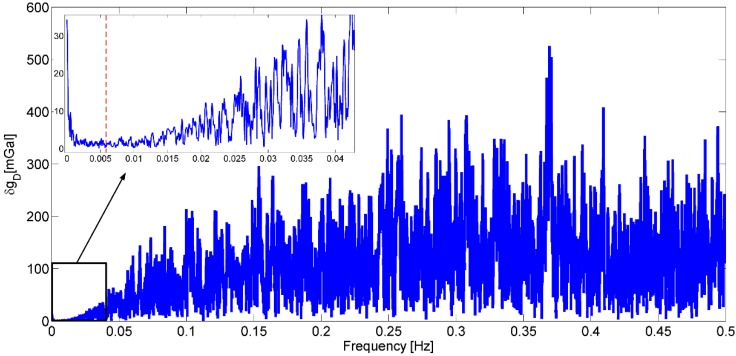
Spectrum of raw gravity measurements. The red dot line shows the cut-off frequency is 0.00625 Hz (correspond to a 160 s length filter).

**Figure 6:**
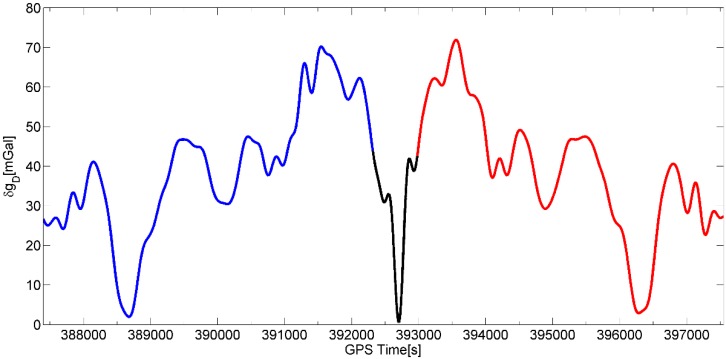
Filtered gravity disturbance along the primary repeated flight A. The northbound flight is shown in blue while the southbound one is marked in red. The black curve between time 392,000 s and 393,000 s represents a 360° turn of the aircraft.

In this study we will only show the first result of gravity disturbance at flight height for the primary repeated line A (bold black line in Figure 3), coincident with the earlier DTU Space flight line. Line B had much stronger turbulence and wind conditions giving some yet unexplained errors in the SGA-WZ results, and line C and D were not repeated, and ground truth data over the mountains very sparse (Figure 3). Gravity estimation from airborne measurements is a complicated procedure since noise in raw gravity disturbance is extremely large and highly dependent on aircraft dynamics which can be seen in Figure 4, Figure 5 and Figure 6. Figure 4 shows the raw gravity disturbance was disturbed by noise. The data rate is 1 Hz. In the unfiltered data, the noise is extremely large which can be up to $10^{5}$ mGal. It also can be seen that the noise in some periods of the repeated line is no less than in the turning period of the aircraft (shown in black between the GPS time 392,000 s and 393,000 s). This explains the rough field conditions mentioned in the first chapter. In fact, there are major wind direction changes which make the aircraft motion more complicate.

The power spectrum of these gravity measurements is given in Figure 5. Much of the noise distributes in the short wavelength of data. The high frequency noise can be caused by the effects of aircraft vibration on the INS and the amplification of GPS system noise when computing acceleration. And the noise in the lower frequency, e.g., below 0.05 Hz, can be from the phugoid motion of the plane. Although SGA-WZ includes a damping system which could, to some extent, absorb the high frequency noise caused by aircraft vibration and motion, the system was insufficient and should be optimized [21]. The obvious way to eliminate these noise effects is a low-pass filter. In this study, according to the resolution of the reference data, a low-pass filter with a cut-off frequency of 0.00625 Hz (correspond to a 160 s length filter) was used. This cut-off frequency is shown by red dot line in Figure 5. It also shows that noise still distributes in the gravity disturbance below this frequency. There is a trade-off between the resolution and accuracy-if a longer length filter is used, the more accuracy but lower resolution result will be obtained.

**Figure 7:**
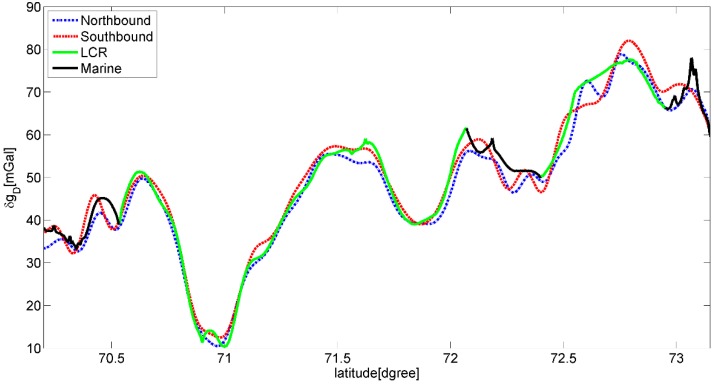
Comparisons of gravity disturbance between the Northbound (blue) and Southbound (red) of the flight A and the former LCR data (green) and marine data (black).

Figure 6 shows the result of profile A after using the low pass filter. Note the black line around the time 392,500 s which represents a 360° turn of the aircraft is close to the largest gravity disturbance in the test line. Comparing the unfiltered and filtered gravity disturbance in Figure 4 and Figure 6, the denoising effect of the used FIR filter is evident. However, not all of noise has been eliminated. As seen in Figure 7 which shows the comparisons between the repeated line and LCR data, a maximal difference between two passes of the repeated line is seen around the north latitude of 72.6°. This is coincident with a major wind direction change at the mouths of rivers (seen in Figure 3). The maximal difference is about 8 mGal found in Table 1 which gives the statistic of comparisons. Since this error appears at the longer wavelength of gravity signal, a more refined filter (e.g., 200 s length of FIR) could possibly reduce the error but lower the resolution. However, the overall repeatability of the SGA-WZ in the two passes of line A is only 1.5 mGal r.m.s., corresponding to approximately 1 mGal for the line error. 

**Table 1:** The statistic of the differences between the repeated line and LCR data (Units: mGal).

Items	Min	Max	Mean	RMS
Northbound-Southbound	−7.5	5.5	−1.7	**1.5**
Northbound-LCR	−7.3	2.6	−1.7	**2.0**
Southbound-LCR	−7.8	4.8	−0.0	**2.6**
Mean(SGA-WZ)-LCR	−7.6	3.1	−0.9	**2.4**

The comparison with the 2001 LCR ground truth data on the same line, including some gap interpolation with marine data (shown in black in Figure 7), shows an r.m.s. comparison of about 3.0 mGal. This discrepancy is consistent with the estimated errors of 2.0 mGal r.m.s. of the LCR airborne gravimetry [26], and thus confirms the estimated survey accuracy of the SGA-WZ.

## 6. Conclusions

The primary repeated flight results of the Greenland test shows that the SGA-WZ gravimeter is capable of delivering results at the 1 mGal r.m.s. accuracy level at 6 km resolution under rough field conditions. This illustrates the potential of SGA-WZ strapdown inertial gravimeter for future airborne gravity surveys, and also implies that SGA-WZ is an appropriate airborne gravimetric system which can be useful for typical geophysical survey activities. The test, however, also shows changes in wind and environmental effects can give higher error levels; research is currently ongoing to locate the sources of these errors, likely linked mainly to the Kalman Filter principles and software implementation.
